# Supplementary material for: Coronary Stent Strut Fractures: Classification, Prevalence and Clinical Associations
Source: J Clin Med. 2021 Apr 19;10(8):1765. doi: 10.3390/jcm10081765 (PMC8072680; doi:10.3390/jcm10081765)
Supplement: Supplementary file 1 [file jcm-10-01765-s001.zip › jcm-1165194-supplementary.pdf]

Comparison with BRS

In 30 cases (40%), multiple fracture patterns were present in the same device. The prevalence of SF was higher in OCTs performed 6 months or more after implantation (53/117 or 45.3%) versus those performed within 6 months from implantation (22/172 or 12.8%, $p < 0.001$ by Chi-Square). Patients with SF had a higher prevalence of acute coronary syndromes at index ($p = 0.013$). Devices with SF were more often implanted in the right coronary artery ($p = 0.021$), in de novo ($p = 0.027$) or in thrombotic lesions (both $p = 0.001$) and in CTOs ($p = 0.042$). Predilatation pressure was lower ($p = 0.013$), device-to-artery ratio was larger ($p = 0.049$), postdilatation was less frequent ($p = 0.025$) in devices with SF. At OCT, segments with SF showed slightly larger average stenosis areas and diameters and a larger degree of asymmetry and eccentricity ($p < 0.001$ for all parameters). The role of device asymmetry and eccentricity was particularly evident for SF patterns 1, 2 and 3 (Supplemental Table S1). The relationship between vessel/device asymmetry/eccentricity and prevalence of SF is presented graphically in Figure 4 and Supplemental Figure S2. Fractures were associated (Table 1 and Figure 5) with malapposition ($p = 0.005$), peri-strut low-intensity areas ($p = 0.001$) and subintimal calcium ($p < 0.001$).

Figure 2 describes the different patterns of SF in the two device types. While the patterns of SF were relatively evenly distributed in DES, pattern 1 was ~four times more frequent in BRS.

Parameters Associated with SF

The results of the uni- and multivariate logistic regression analysis are shown in Supplemental Tables 2B. Implantation of BRS in patients with previous by-pass surgery (OR:17.8 [4.0–79.7], $p < 0.001$), in the RCA (0.47 [0.3–0.8], $p = 0.002$), in calcific lesions (OR:3.5 [2.0–6.1], $p < 0.001$), and the degree of residual stenosis after predilation as well as the predilatation balloon diameter were associated with SF.

Supplemental Table S1. Parameters of device asymmetry and eccentricity. Both eccentricity and asymmetry tended to be consistently larger in devices with fractures, independently of the device type. BVS.

.		Maximal Lumen Asymmetry							Maximal Stent Asymmetry				
		Without		With					Without		With		
		Median	IQR	Median	IQR	*p*-Value			Median	IQR	Median	IQR	*p*-Value
**Fracture**	BVS	0.22	0.11	0.29	0.17	<0.001	**Fracture**	BVS	0.2	0.1	0.24	0.14	<0.001
	DES	0.23	0.1	0.26	0.21	0.02		DES	0.21	0.1	0.26	0.18	0.01
**Pattern 1**	BVS	0.22	0.12	0.26	0.17	0.06	**Pattern 1**	BVS	0.21	0.12	0.23	0.11	0.38
	DES	0.23	0.11	0.37	0.13	0.02		DES	0.21	0.1	0.37	0.16	0.01
**Pattern 2**	BVS	0.23	0.12	0.32	0.23	0.05	**Pattern 2**	BVS	0.21	0.1	0.3	0.21	0.04
	DES	0.23	0.11	0.23	0.13	0.87		DES	0.21	0.1	0.21	0.1	0.84
**Pattern 3**	BVS	0.22	0.11	0.33	0.12	<0.001	**Pattern 3**	BVS	0.21	0.11	0.31	0.21	0.01
	DES	0.23	0.11	0.26	0.1	0.7		DES	0.21	0.1	0.25	0.14	0.46
**Pattern 4**	BVS	0.23	0.12	0.29	0.23	0.05	**Pattern 4**	BVS	0.21	0.11	0.27	0.21	0.2
	DES	0.23	0.11	0.42	0.29	0.05		DES	0.21	0.1	0.34	0.23	0.15
		Minimal Lumen Eccentricity							Minimal Stent Eccentricity				
		Without		With					Without		With		
		median	IQR	median	IQR	*p*-value			median	IQR	median	IQR	*p*-value
**Fracture**	BVS	0.7	0.12	0.63	0.12	<0.001	**Fracture**	BVS	0.75	0.12	0.7	0.11	<0.001

	DES	0.71	0.11	0.67	0.14	0.09		DES	0.76	0.09	0.74	0.15	0.31
**Pattern 1**	BVS	0.69	0.13	0.66	0.12	0.02	**Pattern 1**	BVS	0.74	0.12	0.71	0.12	0.01
	DES	0.7	0.12	0.61	0.16	0.02		DES	0.77	0.09	0.65	0.14	0.01
**Pattern 2**	BVS	0.69	0.13	0.6	0.1	0.01	**Pattern 2**	BVS	0.74	0.12	0.69	0.1	0.03
	DES	0.7	0.13	0.68	0.1	0.83		DES	0.76	0.1	0.79	0.1	0.42
**Pattern 3**	BVS	0.69	0.13	0.62	0.12	0.01	**Pattern 3**	BVS	0.74	0.12	0.7	0.07	0.01
	DES	0.7	0.13	0.72	0.15	0.66		DES	0.76	0.1	0.78	0.11	0.83
**Pattern 4**	BVS	0.68	0.13	0.49	0.25	0.01	**Pattern 4**	BVS	0.73	0.12	0.76	0.12	0.92
	DES	0.7	0.12	0.56	0.39	0.15		DES	0.76	0.1	0.73	0.25	0.56

BVS: bioresorbable scaffolds.

Supplemental Table S2A. Univariate associations of SF with metallic stents.

	Coefficient	SE	*p*
**Patient Characteristics**			
**Age**	0.0049	0.0186	0.7929
**Male**	−0.0164	0.4735	0.9723
**Family History**	−0.3958	0.4478	0.3768
**Diabetes**	−0.2086	0.4491	0.6422
**Hypertension**	−0.1809	0.4772	0.7046
**Hyperlipidemia**	−0.2927	0.4057	0.4707
**Smoking**	0.0674	0.1711	0.6936
**Prior MI**	−0.2716	0.4507	0.5468
**Atrial fibrillation**	−0.0055	0.5857	0.9925
**Valve disease**	0.7633	0.4533	0.0922
**Prior PCI**	0.0499	0.4113	0.9034
**CABG**	−0.4722	1.0750	0.6605
**Reason PCI**	0.0267	0.1516	0.8602
**Lesion/procedural Characteristics**			
**Vessel**	−0.0831	0.2638	0.7527
**ACC/AHA Type**	−0.2824	0.2391	0.2375
**De novo**	−0.1690	0.4783	0.7239
**Thrombus**	0.0519	0.6041	0.9316
**CTO**	0.4559	1.1387	0.6889
**Overlap**	0.4578	0.4342	0.2917
**Bifurcation**	1.3274	0.4959	0.0074
**Predilatation**	1.2677	0.6551	0.0530
**Predil. balloondiameter**	−0.0643	0.1154	0.5775
**Predil. balloon length**	0.0209	0.0580	0.7183
**Predil. balloon pressure**	−0.0113	0.0629	0.8576
**Implantation pressure**	−0.1070	0.0998	0.2839
**Device to artery ratio**	3.0130	1.6816	0.0732
**Postdilatation**	−0.6273	0.4230	0.1381
**Postdil. balloon diam**	−0.9006	0.5613	0.1086
**Postdil. balloon length**	0.0727	0.0800	0.3629
**Postdil. balloon pressure**	−0.1224	0.0838	0.1439
**Final residual stenosis**	−0.0741	0.0563	0.1876
**Calcium > 180°**	1.3585	0.2927	0.0000
**Multivariate analysis**			

Coefficient	SE	*p*	OR	95%CI

**Bifurcation**	1.2507	0.5831	0.0320	3.4928	1.1139 to 10.9523
**Predilatation**	1.3753	0.7074	0.0519		
**Calcium > 180°**	1.3753	0.3031	0.0000	3.9562	2.1841 to 7.1663
**Time from index to OCT**	0.0005	0.0005	0.3291		

PCI: percutaneous coronary intervention; CABG: coronary artery by-pass graft; CTO: chronic total occlusion.

Supplemental Table S2B. Univariate associations of SF – bioresorbable scaffolds.

	**Coefficient**	**SE**	***p***
**Patient characteristics**			
**Age**	−0.0108	0.0117	0.3566
**Male**	0.6641	0.3770	0.0782
**Family History**	−0.3115	0.3008	0.3004
**Diabetes**	−0.5830	0.3653	0.1105
**Hypertension**	0.2319	0.3215	0.4708
**Hyperlipidemia**	−0.2128	0.2778	0.4437
**Smoking**	−0.0768	0.1107	0.4880
**Prior MI**	0.4152	0.3121	0.1834
**Atrial fibrillation**	−0.2695	0.8523	0.7519
**Valve disease**	0.0656	0.3732	0.8605
**Prior PCI**	0.1469	0.2806	0.6006
**CABG**	2.1072	0.6582	0.0014
**Reason PCI**	0.1056	0.1103	0.3382
**Lesion/procedural Characteristics**			
**Vessel**	−0.4648	0.1812	0.0103
**ACC/AHA Type**	0.2636	0.1273	0.0383
**De novo**	0.2907	0.5664	0.6079
**Thrombus**	0.4974	0.3070	0.1052
**CTO**	−1.2799	1.0516	0.2236
**Overlap**	−0.0936	0.3344	0.7797
**Bifurcation**	−0.0534	0.6644	0.9359
**Predilatation**	−0.0232	0.7704	0.9760
**Predil. balloon diameter**	0.1052	0.0494	0.0332
**Predil. balloon length**	0.0413	0.0345	0.2315
**Predil. balloon pressure**	−0.0007	0.0459	0.9880
**Implantation pressure**	0.0874	0.0620	0.1583
**Device to artery ratio**	1.7403	0.8936	0.0515
**Postdilatation**	0.1742	0.2945	0.5542
**Postdil. balloon diam**	−0.1163	0.5352	0.8280
**Postdil. balloon length**	−0.0260	0.0570	0.6480
**Postdil. balloon pressure**	0.0081	0.0574	0.8878
**Final residual stenosis**	−0.0414	0.0409	0.3106
**Calcium > 180°**	0.7898	0.2146	0.0002

**Multivariate Analysis**					
	Coefficient	SE	*p*	OR	95%CI
**CABG**	2.8782	0.7656	0.0002	17.7831	3.9660 to 79.7378
**Vessel**	−0.7570	0.2448	0.0020	0.4691	0.2903 to 0.7578

**ACC/AHA Type**	−0.0142	0.1679	0.9325	0.9859	0.7094 to 1.3701
**Predil. Balloon diameter**	0.1090	0.0497	0.0281	1.1152	1.0118 to 1.2292
**Residual stenosis after predilatation**	0.0134	0.0067	0.0454	1.0135	1.0003 to 1.0269
**Calcium >180°**	1.2420	0.2885	0.0000	3.4627	1.9672 to 6.0951
**Time from index to OCT**	1.0053	1.0037 to 1.0070	0.0053	0.0008	0.0000

Supplemental Table S3. Device failures.

Time From Index to OCT	Sex	Reason for PCI	Vessel nst	Device	Lesion Type	Predilation	Postdilation	Antiplatelet Therapy	Sizing	Fractures	Fracture Type (Most Severe)
**Thrombosis**											
**4**	Male	NSTEMI	LCX	DES	C	yes	No	Clopidogrel, ongoing	1.10	0	0
**262**	Male	UnstAng	LCX	BRS	B2	yes	No	Prasugrel, ongoing	1.12	1	3
**455**	Male	CCS	LCX	BRS	B2	yes	Yes	Clopidogrel, interrupted	1.04	1	3
**0**	Male	CCS	LCX	DES	B2	yes	Yes	Prasugrel	1.17	1	3
**367**	Male	UnstAng	RCA	BRS	B2	yes	No	Clopidogrel, interrupted	1.17	1	3
**690**	Female	NSTEMI	LAD	BRS	A	yes	Yes	Prasugrel, interrupted	1.34	1	4
**690**	Female	NSTEMI	LAD	BRS	A	yes	Yes	Prasugrel, interrupted	1.18	1	4
**635**	Male	STEMI	LAD	BRS	C	No	No	Prasugrel, interrupted	1.42	1	2
**549**	Male	CCS	LCX	BRS	B2	yes	No	Clopidogrel,interrupted	1.54	1	4
**0**	Male	STEMI	LCX	DES	B1	yes	No	Ticagrelor	0.86	1	1
**0**	Female	NSTEMI	RCA	BRS	B1	yes	Yes	Prasugrel	1.30	0	0
**0**	0	STEMI	RCA	BRS	C	yes	Yes	Prasugrel	1.02	1	3
**578**	Male	STEMI	RCA	DES	A	yes	Yes	Ticagrelor, interrupted	1.20	1	4
**0**	Male	CCS	RCA	BRS	B2	yes	Yes	Prasugrel	1.08	0	0
**762**	Male	CCS	LCX	BRS	B2	yes	Yes	Ticagrelor, interrupted	2.00	1	3
**762**	Male	CCS	LCX	BRS	B2	yes	Yes	Ticagrelor, interrupted	1.59	1	3
**0**	Male	STEMI	LAD	DES	B1	yes	No	Prasugrel	1.18	0	0
**0**	Male	STEMI	LAD	DES	B1	yes	No	Prasugrel	1.11	0	0
**0**	Male	STEMI	RIVA	DES	B2	yes	No	Ticagrelor	1,42	0	0
**145**	Male	NSTEMI	RCX	DES	B2	yes	no	Clopidogrel	1,38	1	3
**Restenosis**											
**600**	Male	CCS	LCX	DES	B2	yes	No	Prasugrel	0.84	1	1
**630**	Male	CCS	LAD	DES	B2	yes	No	Prasugrel	0.83	1	2
**294**	Male	UnstAng	LCX	BRS	C	yes	Yes	Prasugrel	0.87	1	1
**374**	Male	CCS	RCA	BRS	B2	yes	No	Prasugrel	1.20	0	0
**529**	Male	CCS	LCX	BRS	C	yes	Yes	Prasugrel	1.31	1	1

**790**	Male	UnstAng	LAD	BRS	B1	yes	No	Clopidogrel	1.81	1	1
**390**	Male	STEMI	RCA	BRS	C	yes	No	Prasugrel	1.02	1	4
**194**	Male	CCS	LCX	BRS	B1	yes	Yes	Prasugrel	1.28	1	1
**469**	Female	NSTEMI	LCX	BRS	B2	yes	Yes	Prasugrel	1.63	1	4
**778**	Female	CCS	RCA	BRS	A	yes	Yes	Prasugrel	1.78	0	0
**778**	Female	CCS	RCA	BRS	A	yes	Yes	Prasugrel	1.70	0	0
**226**	Male	NSTEMI	LAD	BRS	C	yes	Yes	Prasugrel	1.15	1	2
**863**	Male	NSTEMI	LAD	BRS	A	yes	Yes	Prasugrel	1.45	1	1
**180**	Female	CCS	LCX	DES	B1	yes	No	Clopidogrel	1.50	1	1
**180**	Female	CCS	LCX	DES	B1	yes	No	Clopidogrel	1.41	1	4
**300**	Male	CCS	LCX	DES	A	yes	Yes	Clopidogrel	1.15	1	1
**371**	Male	STEMI	LCX	DES	B2	yes	Yes	Ticagrelor	1.01	0	0
**360**	Male	CCS	LAD	BRS	A	yes	Yes	Clopidogrel	1.24	0	0
**188**	Male	CCS	RCA	DES	B2	yes	no	Prasugrel	1.50	0	0
**168**	Male	CCS	RCA	DES	B2	yes	no	Prasugrel	1,74	1	4
**18**	Female	CCS	LAD	DES	B2	yes	no	Ticagrelor	1,48	0	0
**144**	Male	CCS	RCA	DES	B2	yes	no	Clopidogrel	1,21	1	3
**188**	Male	CCS	RCA	DES	B2	yes	no	Clopidogrel	1,21	1	4
**257**	Female	CCS	LAD	DES	B2	Yes	Yes	Clopidogrel	1.34	1	4
**814**	Male	CCS	LAD	DES	B2	Yes	Yes	Clopidogrel	1.05	0	0
**106**	Female	CCS	LAD	DES	B2	Yes	Yes	Clopidogrel	1.18	1	1
**0**	Male	CCS	RCA	DES	B2	Yes	no	Clopidogrel	1.37	1	3
**192**	Male	CCS	RCA	DES	B2	Yes	Yes	Prasugrel	1.83	1	3
**805**	Male	CCS	RCX	DES	B2	yes	Yes	Clopidogrel	1.25	0	0
**0**	Male	CCS	RCA	DES	B2	yes	No	Ticagrelor	1.49	1	3
**187**	Male	CCS	LM	DES	B2	yes	No	Clopidogrel	1.41	1	0
**780**	Male	UnstAng	RIVA	DES	B2	yes	No	Prasugrel	1.15	0	0
**188**	Male	CCS	RCA	DES	B2	yes	No	Clopidogrel	1.38	0	0
**0**	Male	CCS	RCA	DES	B2	yes	No	Clopidogrel	1.16	1	4

LCX: left circumflex; LAD: left anterior descending; CCS: chronic coronary syndrome. Sizing: ratio of device to artery.

Supplemental S4A. A Multivariate analysis of the predictors of device failure–metallic stents.

	Odd´s Ratio	CI	*p*
**Thrombus**	1.4	0.4 to 5.1	0.61
**Fracture**	12.5	5.3 to 29.4	0.0000

Supplemental Table S4B. Multivariate analysis of the predictors of device failure–bioresorbable scaffolds.

	Odd´s ratio	CI	*p*
**Fractures**	7.2	2.2 to 23.2	0.0010
**Smoking**	1.3	0.9 to 1.8	0.1559
**Prior PCI**	2.0	0.7 to 5.8	0.2212
**Malappositions**	0.2	0.02 to 1.6	0.1303
**Calcium >180°**	1.3	0.7 to 2.8	0.4178
**Device-to-artery ratio**	202.8	17.0 to 2413.8	0.0000

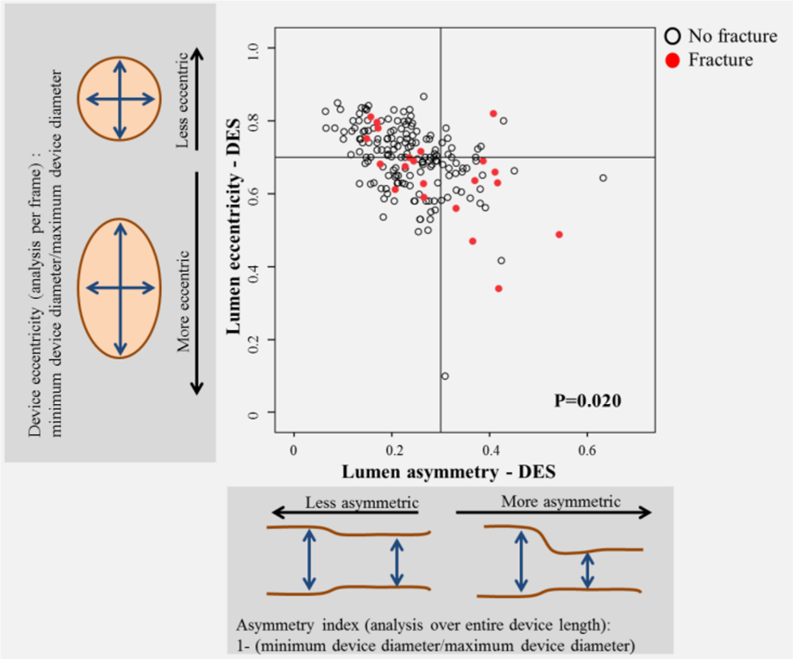

		No fracture		Fracture		p-value
Lumen AI < 0,3	Lumen EI > 0,7	82	94.3%	5	5.7%	0.020
	Lumen EI < 0,7	50	87.7%	7	12.3%	
Lumen AI > 0,3	Lumen EI > 0,7	10	90.9%	1	9.1%	
	Lumen EI < 0,7	22	73.3%	8	26.7%	

Supplemental Figure S1. Asymmetry and eccentricity for drug eluting stents.

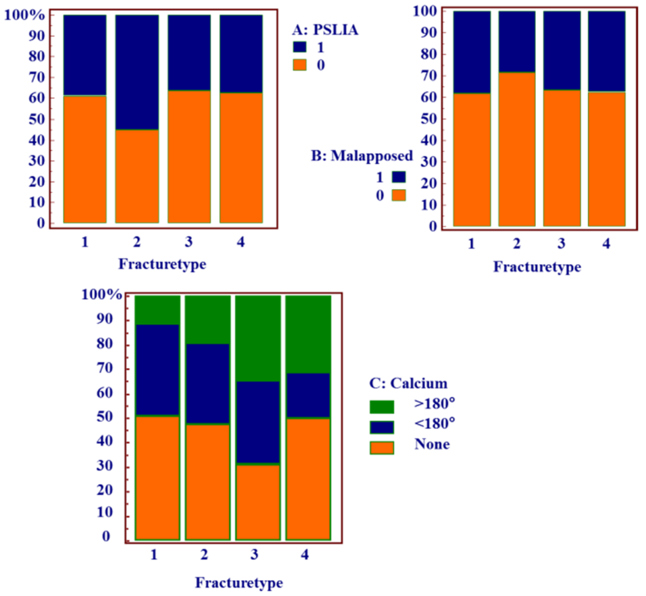

Supplemental Figure S2. Incidence of peri-strut low intensity areas (PSLIA, A), malapposition (B) and calcium (C) by fracture type.

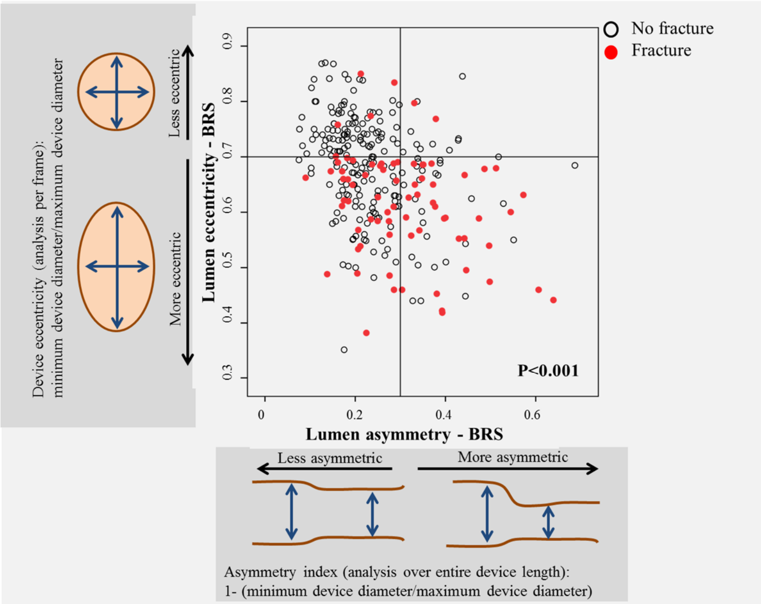

		No fracture		Fracture		p-value
**Lumen AI < 0.3**	**Lumen EI > 0.7**	96	95.0%	5	5.0%	**<0.001**
	**Lumen EI < 0.7**	80	69.0%	36	31.0%	
**Lumen AI > 0.3**	**Lumen EI > 0.7**	11	84.6%	2	15.4%	
	**Lumen EI < 0.7**	27	45.8%	32	54.2%	

Supplemental Figure S3. Asymmetry and eccentricity in scaffolds

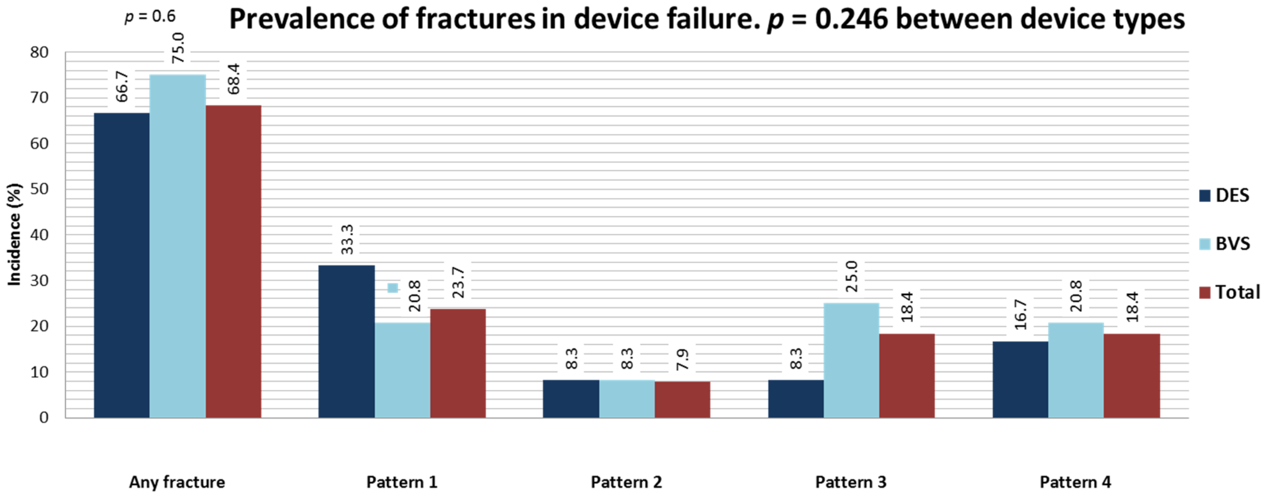

Supplemental Figure S4. Prevalence of fractures in device failure
